# Physical Activity and Body Image Perceived by University Students during the COVID-19 Pandemic: A Systematic Review

**DOI:** 10.3390/ijerph192416498

**Published:** 2022-12-08

**Authors:** Eliane A. Goicochea, Bruno Coloma-Naldos, Jeel Moya-Salazar, Víctor Rojas-Zumaran, Jeel G. Moya-Espinoza, Hans Contreras-Pulache

**Affiliations:** 1 School of Medical Technologist, Faculties of Health Science, Universidad Tecnológica del Perú, Lima 51001, Peru; 2 School of Biomedicine, Faculties of Engineering, Universidad Tecnológica del Perú, Lima 51001, Peru; 3 South American Center for Research in Public Health and Education, Universidad Norbert Wiener, Lima 51001, Peru; 4 Pathology Department, Hospital Nacional Docente Madre Niño San Bartolomé, Lima 51001, Peru; 5 Qualitative Unit, Nesh Hubbs, Lima 51001, Peru

**Keywords:** physical activity, body image, COVID-19 pandemic, university students

## Abstract

Our objective was to assess the perception of body image and physical activity in university students during the COVID-19 pandemic. Of 74,270 papers found on 13 search engines between 12 August 2020, and 2 November 2021, we identified six studies (n = 1392 and 1097 were women). We found several results on the perception of both variables during the pandemic. First, physical activity tended to decrease or have negative changes, either because they dedicated less time to it, decreased the type of intensity or because they dedicated more time to sedentary activities. In addition, women were more physically active than men, since men perceived a decrease in their levels of physical activity. Secondly, there were also slight changes in the perception of body image; several students perceived that they gained weight and others had an increase in their BMI. It is even noted that those who were physically active or who were older in the population studied had a better perception of their actual physical condition. Similarly, we found that a concern for body image and even negative changes in the perception of appearance during confinement were reported. In conclusion, we found changes in the perception of physical activity and body image in college students during the COVID-19 pandemic.

## 1. Introduction

Physical activity levels are an important factor for health, as they help prevent chronic non-communicable diseases, which in the last two decades have increased mortality in young adults [1]. The World Health Organization (WHO) reports that at least 1/4 of adults do not get the recommended amount of physical activity (minimum of 150 min of moderate-intensity physical activity per week or 75 to 150 min of vigorous-intensity physical activity) [2].

College students may be more prone to low physical activity due to their academic pursuits, lifestyles, and work activities [3,4]. As with college students, there are different situations that condition physical activity levels; it was observed that in Rome university women, 34% were sedentary and 33% had poor physical fitness [5]. This is contrasted in Europe; a Spanish study shows that 74.5% were physically active in some way [6]. Other studies in Americans have shown that almost half of college students do not reach the recommended levels of physical activity [4]. In Latin America, low levels of physical activity have been reported in Peruvian universities that increased dramatically as the level of education increased [7], while a study based on surveys in Colombian universities showed a 70% frequency of physical activity [8].

On the other hand, the level of physical activity is significantly related to the assessment of self-perceived body image; in fact, in young adults, a meta-analysis of 12,519 young people has described a positive relationship between physical activity and body image, mainly among men. This is because there is some social anxiety about physique, specifically about muscularity or thinness, which drives males to engage in regular physical activity to stay fit [9]. While physical appearance and excessive body weight have a great impact, one of the determining factors on the self-perception of body image is the psychological aspect, which would come to be the emotional state and its social environment. For instance, in the United Kingdom, it was shown that female students perceived themselves as “too fat”. This dissatisfaction correlates with poor weight control practices, as they may develop eating disorders, which implies a deterioration in the quality of life [10]. Data from a study in India showed that female university students with low satisfaction with body image have a high probability of having low levels in factors associated with quality of life, such as low self-esteem, low life satisfaction, and feelings of inferiority, and are exposed to a higher risk of depression or anxiety [11]. Similarly, in Peru it has been shown that body image distortion has a negative effect on the subjective well-being of female university students [12], while other studies indicate that affective images and excessive pressure for physical appearance affect overall body image satisfaction [13,14].

In addition to those already mentioned, there are more characteristics of university students that are related to physical activity. For example, physical activity tends to decrease due to demanding schedules, and there is an increase in caloric intake when leaving, for the most part, the family environment. In this sense, it should be considered that university students are a group with complex and peculiar characteristics as they are in a critical period of their lives. There are habits that make college students a peculiar group that, in many cases, cause their physical activity levels to decline (i.e., increased sitting and studying hours, increased access to unhealthy foods, alcohol and tobacco consumption, etc.) [15].

This whole context has undoubtedly been affected by the COVID-19 pandemic. The restrictions imposed to prevent the spread of the disease to the distance education modalities have affected university welfare [16,17,18]. It has recently been shown that sedentary levels increased during confinements with the persistence of sedentary behaviors [19]. Body image may also be affected by the pandemic, in fact, an extreme preoccupation with body image was demonstrated in 65% of Colombian university students [20]. In addition to the risk habits that have increased during the pandemic, it is possible that both distance education and remote work contribute to a low level of physical activity, as it forces people to spend more time at rest while working [21,22,23]. This low physical activity may have an impact on the perception of body image satisfaction and the overall well-being of university students.

In this study, we conducted a systematic review to determine the physical activity and body image perceived by college students during the COVID-19 pandemic. We hypothesized that (1) the COVID-19 pandemic may affect physical activity due to mobility restrictions and quarantine to prevent the spread of the disease; and (2) body image can be altered since a sedentary lifestyle and weight gain have been frequent factors during the lockdown. For this reason, this review is undoubtedly important, as body image is a key factor in college students’ well-being, as it affects perceptions of the body and is related to the physical activity they perform.

## 2. Materials and Methods

### 2.1. Study Design, Data Sources and Search Strategy 

This review follows the reporting guidelines specified in the Preferred Reporting Items for Systematic Reviews and Meta-Analysis (PRISMA) 2020 [24] and the protocol of this systematic review has been registered on the International Prospective Register of Systematic Reviews (PROSPERO) with code: CRD42022359284. We searched seven databases (PubMed, Scopus, Web of Science, EMBASE, Scielo, Latindex, LILIACS and EBSCO), four public prepublication servers (medRxiv, bioRxiv, SportRxiv and Preprints), Google Scholar and Alicia CONCyTec (Peruvian Thesis Repository) from 24 April 2022 to 8 May 2022.

The database search strategy was carried out using Boolean descriptors with a combination of keywords and subject headings. We identified publications using the terms ((Motor Activity OR Physical activity) AND Body Image AND COVID-19) and the corresponding translations into Spanish and Portuguese. The manual search was performed in the reference lists of included studies between 12 August 2020 and 2 November 2021, when studies meeting the inclusion criteria were identified.

### 2.2. Inclusion and Exclusion Criteria 

**Figure 1:**
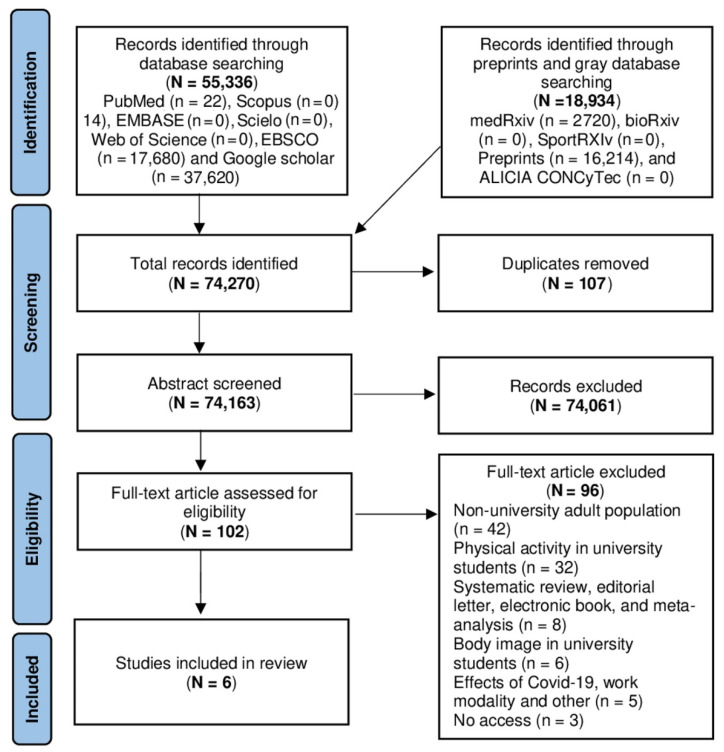
PRSIMA flowchart of the studies.

Included studies met the following criteria: (i) male and female university students over 18 years of age; (ii) studies conducted during the COVID-19 pandemic; and (iii) articles in English, Portuguese, and Spanish. Studies conducted in other populations (infants, pregnant women, older adults, people with physical disabilities or pathologies that alter their physical activity); and studies conducted prior to the COVID-19 pandemic were excluded. In addition, systematic reviews, meta-analyses, letters to the editor, history articles, reflection articles, case reports, recommendation articles, guidelines, and protocols were eliminated (Figure 1).

### 2.3. Screening Study, Data Extraction and Analysis 

Abstracts were independently screened by three authors (EAG, BC-N and JM-S) and excluded if they did not meet the inclusion criteria. Under the defined protocol, the three authors also reviewed the full text for inclusion in the final analysis. The authors resolved disagreements by consensus at each stage of the review. A correlational analysis was performed to determine overall inter-reviewer agreement using Cohen’s Kappa correlation test. 

### 2.4. Data Extraction, Quality Assessment and Data Analysis 

**Figure 2:**
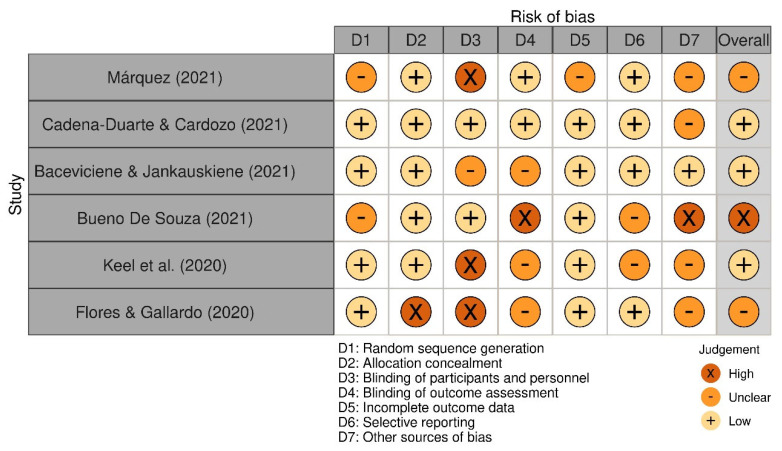
Bias assessment of the study included in this review. N = 6 [25,26,27,28,29,30].

Data extraction was performed from the data matrix in MS-Excel 2013 (Microsoft Corp., Redmond, WA, USA) using the CASPe (Critical Appraisal Skills Programme) template to capture the desired information from the systematic reviews. Bias assessment was developed using the Cochrane risk of bias tool (Robvis 2.0), and studies that did not contribute to the study objective (analyzing at least one variable) were considered at high risk of confounding (Figure 2). Disagreements were resolved by consensus among the authors. The descriptive analysis of the included studies was developed in IBM SPSS version 23.0 (Armonk, NY, USA) estimating frequencies for categorical data and the estimation of measures of central tendency for continuous data.

## 3. Results

After searching the database in 13 servers we found a total of 74,270 records, 37,620 from Google Scholar, 17,680 from EBSCO, 16,214 from Preprints, 2720 from medRxiv, 22 from PubMed, and 14 from Scopus. We did not find any studies in Latindex and LILIACS. Applying the exclusion criteria and eliminating duplicate articles, we obtained 102 articles, and then evaluated them strictly on the basis of the inclusion criteria, eliminating 96 of them and ending with six articles that were included in the systematic review (Figure 1). Of the total number of articles included, five were conducted in Latin America, specifically in Colombia, Peru, and Brazil; and one was conducted in the United States and Europe. The total enrollment of the six studies was 1392 participants, and 1097 were women.

### 3.1. Characteristics of the Studies

The six studies evaluated the two research variables (physical activity and self-perceived body image) and their changes during the COVID-19 pandemic in the university population, in different phases at some point during the pandemic, with different approaches and instruments. During the COVID-19 pandemic, changes in physical activity and body image have emerged. With this, we will go on to detail the characteristics of the studies included in the review. 

The study by Márquez et al. [25] was conducted on 16 university students (15 women) in the Nutrition and Dietetics career in Bogota, Colombia. To evaluate physical activity, four items of a questionnaire of their own were used, from which it was obtained that the number of students performing physical activity before and during the pandemic was maintained; however, the time dedicated to it decreased, so they did not comply with the recommendations set forth by the WHO. In the same country, the study by Cadena-Duarte et al. [26] consisted of 499 university students from a private institution in Bogota and used the self-administered Physical Self Questionnaire Physical Self Questionnaire (PSQ) Spanish adapted version consisting of 36 items to assess the physical activity dimension as the perception of strength and competence, obtaining better scores in younger women (≤23 years) and men between the age of 24 to 29 years. In contrast, when comparing the perception of body image between age groups there was a better perception in the older ones compared to the younger ones (Table 1).

The study by Baceviciene et al. [27] was conducted in Lithuania and involved 230 university students of both genders aged 19–39 years old, the majority of whom were women (182 women and 48 men). They were surveyed before and during the pandemic with the Leisure Time Exercise Questionnaire (LTEQ) to determine their levels of physical activity, finding that men had significantly decreased their practice compared to women. On the other hand, using the Lithuanian version of the Multidimensional Body Self Relations Questionnaire-Appearance Scales (MBSRQ-AS), they observed that during quarantine, women indicated a slight increase in Body Mass Index (BMI) and in satisfaction with appearance, while in men there was no significant difference.

In the study by Bueno de Souza et al. [28] on female students at the Midwest State University in Brazil, they applied their own questionnaire showing that, out of 294 participants, 273 were undergraduate students. According to their results regarding body image, they explain that the majority reported negative changes during social isolation and, regarding the practice of physical activity, physically active university students indicated positive appearance changes with statistical significance.

The cross-sectional study by Keel et al. [29], in 90 psychology students at Florida State University, had a mean age of 19 years, and 79 of the participants were female. They applied two evaluations, the first in January 2020, when COVID-19 had not yet reached pandemic status and the universities were operating normally, and the second in April 2020, when the restrictions on attendance due to the pandemic came into effect. In the second evaluation, questions were added to assess the impact of COVID-19 on participants’ lives. Comparing the results between the two times, we found that there were perceptions of weight gain and decreased physical activity, as well as a significant change in weight perception by frequently being in a higher weight category.Despite this, there was no significant change in actual body mass index between the two times.

In the Peruvian study by Flores et al. [30], conducted with 263 students (18 to 23 years old) from 15 professional careers, they evaluated physical activity using the International Physical Activity Questionnaire (IPAQ) translated into Spanish in its short version, with the finding that they performed light exercise more frequently, with an average of 6.48 h per week. With respect to body image, they used the Body Shape Questionnaire (BSQ) to evaluate the emotions related to this variable and the intensity of the interest given to it. They reported that most of the participants have no concern about their body image (47.2%), followed by those that had a slight concern (27.4%). 

### 3.2. Physical Activity

The similarity that stands out in the six articles is that the populations studied tend to be mostly women, and two of them have been conducted in this population only. Regarding this group, in the study by Flores et al. [30], it was found that most of the participants do not perform physical activity as part of their routine, light physical activity being the most practiced. On the other hand, Bueno de Souza et al. [28] in their study divided their participants into two groups: active (68.7%) and inactive (31.3%), and after analyzing the perceived changes, physically active university students indicated positive changes in appearance with statistical significance. 

The studies by Marquez et al. [25] and Keel et al. [29] had participants of both genders but from a professional undergraduate career. Marquez et al. [25] evaluated physical activity levels in undergraduate nutrition students with a questionnaire that had questions about their perceptions before and during the pandemic. They perceived that before the pandemic, 68.8% of the students were physically active, and currently 68.7% were physically active. According to the minutes per day, 50% performed 150–300 min per week before the pandemic, and currently, only 25% perform physical activity during that time, failing to reach the recommendations provided by the WHO. On the other hand, Keel et al. [29] divided their study into two moments evaluating psychology students, and found that among the changes perceived since the beginning of the pandemic there was a decrease in physical activity associated with an increase in sedentary behaviors, specifically the spending of more time watching television. 

The study by Baceviciene et al. [27] found that male university students significantly decreased their levels of physical activity (from a mean of 78.77 to 58.77 points), while in women no significant changes were observed in this variable during lockdown. On the other hand, Cadena-Duarte et al. [26] divided their participants into four groups according to age ranges and found that the perception of the physical activity dimension based on perceived strength and competence reflected better scores in younger women aged 23 years or younger and, in the case of men, it was those in the second category with a range of 24 to 29 years old (Table 2).

Finally, most studies have shown changes in physical activity during the pandemic, reducing the time and intensity of exercise, and differentially affected by gender and age group.

### 3.3. Body Image

Most studies used questionnaires with qualitative responses for the description of self-perceived body image. The instrument used by Flores et al. [30] showed that most of the female students had no concern about their body image (47.2%), followed by those with mild concern (27.4%), moderate concern (16.7%) and extreme concern (8.7%). However, in the study by Bueno de Souza et al. [28] it was found that 149 (50.7%) university women reported negative changes, 105 (35.7%) reported no changes, and only 40 (13.6%) of them reported positive changes.

The study by Márquez et al. [25] used Body Silhouettes by Stunkard & Stellar (1990) for the perception of body image [31] of students of both sexes at the nutrition school. Forty-three percent of the students perceived themselves as normal weight (Silhouette 6) and 25% perceived themselves as obese (Silhouette 7). On the other hand, Keel et al. [29] found no significant changes in reported weight at Time 1 (mean (SD) = 140.81 ± 28.92 pounds) and reported weight at Time 2 (141.17 ± 27.50 pounds; *p* = 0.57) or BMI from Time 1 (22.93 ± 4.02 kg/m^2^) to Time 2 (22.91 ± 3.70 kg/m^2^; *p* = 0.89). Although there was no statistically or clinically significant change in reported weight or BMI, there was a significant increase in the mean value for weight description from Time 1 to Time 2 (*p* = 0.03).

The study by Baceviciene et al. [27] found that, during quarantine, no changes in BMI or body image were observed in men. In women, however, BMI (from a mean of 21.83 to a mean of 22.02) and satisfaction with body appearance (from a mean of 3.29 to a mean of 3.38) increased significantly. In contrast, Cadena-Duarte et al. [26] in their study, when dividing the participants into age groups, found that in both sexes the perception of body image was better in older university students in contrast to younger ones, being more noticeable in males between 24 and 36 years old (Table 3).

Finally, our results show slight changes in body image among college students during COVID-19; only one study showed a negative effect, and many studies found no body image problems, although differences between genders were found.

## 4. Discussion

This systematic review contains papers from three continents, including a total of 1392 university students, and demonstrates that both self-perceived body image and physical activity in this population have been affected by confinement during the pandemic. On the one hand, with regard to body image, their perception has been altered mostly negatively, and on the other hand, physical activity has been reduced. In addition to these studies, there were also reports that were conducted pre- and post-pandemic, demonstrating that body image and physical activity in university students has changed. 

Among the strengths is that this review is the first study specifically on the variables of physical activity and self-perceived body image during the COVID-19 pandemic in college students. There are studies that have evaluated these variables in Latin American countries independently before the pandemic. In Mexico, for example, research showed that a lack of physical activity was associated with perceived overweight or obesity in 7 out of 10 citizens [32]. Furthermore, in Colombia, it has been observed that physical activity levels decrease with advancing age and university age [8]. The same was observed in the Peruvian population in general [7]; although this is contrasted with another study in Peruvian university students in the first year of the faculty of health sciences, where 50% performed moderate intensity physical activity and 11.1% vigorous activity [33].

Among the studies conducted during the pandemic specifically in the female population, we see that, in the population of Flores et al. [30], most do not perform sufficient physical activity as part of their routine, light exercise being the most practiced type of physical activity. The author considers these results to be common, since this population is characterized by fulfilling many academic responsibilities in short periods of time, the involvement in extracurricular activities, and interests appropriate to their age. This is significant since the relationship between this variable and changes in appearance is significant, as shown by Bueno de Souza et al. [28], who had 68.7% of physically active students and after analyzing the perceived changes, this group was the one that indicated positive changes in appearance during the pandemic. These results may be due to different motivations of the participant, such as having good health and physical attractiveness. The link between physical activity and health had been established before the pandemic. In this sense, a study of six Polish universities demonstrated this relationship, with significant differences between the types of physical activity performed by men and women [34]. 

Continuing with those studies that observed the changes produced in both sexes, Márquez et al. [25], in a study of 16 students, showed that 68.8% and 68.7% were physically active before and during the pandemic, respectively. However, the time dedicated to physical activity was reduced, as before the pandemic 50% performed 150–300 min per week, and after the pandemic only 25% dedicated an amount of time within this range. These results are detrimental because they do not comply with WHO recommendations to prevent the onset of chronic non-communicable diseases, highlighting the importance of healthy habits during this formative stage, which are usually maintained in the rest of adulthood. Similarly, Keel et al. [29] found in their sample that since the beginning of the pandemic there was a decrease in physical activity associated with an increase in sedentary behaviors. Baceviciene et al. [27] found that male university students significantly decreased their levels of physical activity, whereas females did not demonstrate these changes. In addition, in both sexes, an increase in Internet surfing time was seen during confinement. Little physical activity may lead to a decrease in the quality of life during the pandemic, as has been reported in Chilean university students [35]. 

Another study that reflects the impact of isolation due to the pandemic is that of Cadena-Duarte et al. [26]. For their group of students, the perception of physical activity decreased based on the dimensions of strength and perceived competence of the instrument used in their research. In this sense, it should be considered that the impact of the pandemic and the measures taken to combat it are not yet fully calculated, even less so in a dynamic and modern space such as the life of university students within the “new normal” [36]. In relation to this, a systematic review of the lifestyles of university students carried out before the pandemic found that there is a great tendency to unhealthy habits in this sector of the population (i.e., low frequency of physical activity, sedentary attitudes). On the other hand, although body image itself is not included among the variables of the review, it does include other psychological factors, such as stress, anxiety, depression, and low self-esteem, which may be associated with the distortion of the perception of body image. In this sense, it is feasible to suggest that these psychological conditions that are normally present during university life, added to the stress produced by the social distancing measures due to the pandemic, could be factors that directly contribute to the distortion of body image perception in students [37].

On the other hand, body image has been evaluated with various instruments including anatomical silhouettes that have an approximate BMI depending on which one is chosen. In studies that applied this instrument prior to the pandemic, it was observed that women tend to perceive themselves as more obese than they really are [38]. Similarly, another study that compared two groups of female students from Mexico and Canada had more cases of overweight and obesity. Between the two groups, an average of 54.3% of students were dissatisfied with their body image and wished to be thinner [39]. Therefore, this phenomenon or overweight effect, happens frequently during the college life of female students.

In the studies conducted during the pandemic only in the female population, we see that Flores et al. [30] found that at least 52.8% felt some degree of concern about their body weight, while 27.4% of the sample felt a slight concern. This is relevant since these results are associated with an increased risk of developing eating disorders. An explanation could be supported by the study by Bueno de Souza et al. [28], which has shown that under the requirement to comply with beauty standards, 50.7% of university students reported negative changes, being the most affected by the lockdown. 

Regarding the studies that had participants from a particular career, the study by Márquez et al. [25], which was made up of nutrition and dietetics students (mostly women) (94%), showed that 43.8% of students of both sexes perceived themselves as having a normal weight, while 25% were obese despite having healthy eating characteristics (increased consumption of fruits and vegetables and decreased consumption of foods). It showed that 43.8% of students of both sexes perceived themselves as having a normal weight, while 25% perceived themselves as obese even though they had healthy eating characteristics (increased consumption of fruits and vegetables and decreased consumption of fast food). In addition, 87.5% had a normal BMI, so considering that most of them were women, the tendency to overestimate overweight is understandable. However, in the sample of Moroccan students from the faculty of science and technology, it was highlighted that there was a tendency to underestimate body weight, as 52.9% perceived themselves as having a normal weight and only 4.2% perceived themselves as obese, the latter percentage being approximately half of the students who actually had some degree of obesity. Likewise, regarding body dissatisfaction and intentions to modify their weight in this sample of students, it was found that 69.8% were dissatisfied with their weight, of which 70.3% expressed desires to weigh more. In the Moroccan population, there is traditionally a preference for a heavier curved body and there is a tendency to desire to gain weight with age, especially in women [40]. These differences between results show how much the perception of weight and body image can vary among students.

This situation may be exacerbated during the pandemic, as Keel et al. [29] found that although there were no statistically significant changes in reported weight or BMI a significant increase in the mean value for weight description during the pandemic (*p* = 0.03) can be seen. These alterations in weight perception occur because more than 60% of participants had increased concerns about weight, shape, and eating habits since the onset of COVID-19. This is negatively associated with the situation of anxiety, isolation, and frustration brought about by the pandemic. This is confirmed in the study by Baceviciene et al. [27], whereby the participants for the most part coped well with the situation of confinement. The author, when dividing the students by sex, observed that the BMI or body image without changes during the pandemic was limited to men, since in women the BMI significantly increased (from a mean of 21.83 to a mean of 22.02) as did satisfaction with body appearance (from a mean of 3.29 to a mean of 3.38).

On the other hand, Cadena-Duarte et al. [26], when dividing the participants into age groups, found that in both sexes the perception of body image was better for older participants, especially in males between 24 and 36 years of age, in contrast to younger ones. Studies indicate that there is a better perception of image and physical attractiveness, especially in males, as age advances because the importance of appearance is attenuated. 

Similarly, in new college students between 17 and 19 years of age, the relationship between perceived body image and BMI is highly significant (*p* < 0.001) [41]. This is because these variables were seen to be highly related during pre-pandemic times regardless of age. For example, in a meta-analysis conducted before the pandemic on the association between body image and physical activity in men, it was reported that body image is positively associated with physical activity, since engaging in any type of physical activity can have a positive impact on body image and has a greater impact on adolescents and college students. These results contrast with one of the key points made in this review, as the positive association between body image and physical activity is less significant during the pandemic, due to the increased distortion of body image perception experienced by the college population in these times, regardless of age [9].

The implications on body image that we have found are that male university students tend to perceive themselves as physically better, while female university students tend to perceive themselves as fatter than they really are. This has implications for their mental health, since it is related to a fear of being overweight as well as body dissatisfaction. This is important to consider, as social isolation due to the COVID-19 pandemic has given way to psychological implications such as anxiety, stress, and depression [17]. Confinement also brought changes in lifestyles, such as increased sedentary behaviors, including spending more time at home, which is associated with a generally negative body image perception, so it is recommended to encourage physical activity in college students to avoid repercussions such as overweight, obesity, or the onset of chronic noncommunicable diseases [42].

## 5. Conclusions

In conclusion, we found different perceptions of physical activity and body image among university students during the COVID-19 pandemic. The results of physical activity undertaken by students show changes during the pandemic. In all the studies, physical activity tends to decrease or have negative changes either because they dedicate less time or decrease the intensity of physical activity, or because they dedicate more time to sedentary activities. There were also changes in the perception of body image, as several students perceived that they gained weight, and others had an increase in BMI. These results are related in that during the pandemic both variables were affected by the change in the daily routine of university students. 

With such varied results, it is important to continue to conduct studies that evaluate the post-pandemic situation because as measures of isolation and social distancing decrease, there will be greater mobility in the population, and it will be possible to directly evaluate the participants. This will allow for the better estimation of whether the perception of body image is really altered, since the studies in this systematic review used silhouettes with an approximate BMI or questionnaires on body satisfaction. Likewise, due to the significant changes in the levels of physical activity, it is a priority to create spaces with adequate biosecurity measures for university students to perform the physical activity while maintaining distance and complying with the sanitary measures established to prevent the spread of COVID-19 in order to avoid the development of non-communicable metabolic-chronic diseases during confinement.

## Figures and Tables

**Table 1:** Baseline characteristics of the included studies.

Year	Author	Country	Study Type	Population	University Students
2021	Márquez [25]	Colombia	Cross-sectional observational	16	Nutrition and Dietetics
2021	Cadena-Duarte. & Cardozo [26]	Colombia	Quantitative approach, in a comparative and cross-sectional study	499	Occupational health and safety administration, systems engineering, industrial engineering, physical education, electronics, graphic communication, computer science, marketing management, business logistics, social work
2021	Baceviciene & Jankauskiene [27]	Lithuania	Observational according to STROBE guidelines for cohort studies	230	No career specified
2021	Bueno De Souza [28]	Brazil	Descriptive quantitative survey	294	Women only: Undergraduate (273) and Graduate (21)
2020	Keel et al., [29].	USA	Longitudinal	90	Psychology
2020	Flores & Roman Gallardo [30]	Peru	Quantitative observational, correlational	263	Women only: Psychology, Industrial Engineering, Administration, Law, Education, Obstetrics, Accounting, Communication Sciences, Medicine, Mechatronic Engineering, Civil Engineering, Advertising, Nursing, Architecture, Dentistry.

**Table 2:** Physical Activity in university students during the COVID-19 pandemic.

Author (Year)	Country	Population	Instrument	Result
Márquez (2021) [25]	Colombia	16	Four items of a self-reported questionnaire on their perception of physical activity before and during the pandemic	Before and during the pandemic, no significant changes were seen in the physical activity of the students since they continued to do it. However, with respect to the time dedicated to physical activity, from 50% who were active for 150 to 300 min per week, it was reduced to 25%.
Cadena-Duarte & Cardozo (2021) [26]	Colombia	499	Physical Self Questionnaire (PSQ) adapted to the Spanish context, modified version by Moreno and Cervelló (2005)	Based on perceived strength and competence, there were better scores in younger women aged 23 years or less and in men aged 24 to 29 years.
Baceviciene & Jankauskiene, (2021) [27]	Lithuania	230	Leisure Time Exercise Questionnaire (LTEQ)	In contrast to women, men significantly decreased their physical activity levels during confinement.
Bueno De Souza (2021) [28]	Brazil	294	Own elaborated questionnaire	The majority of female university students were physically active and indicated positive appearance changes with statistical significance.
Keel et al., (2020) [29]	USA	90	Two items from the Body subscale of the Body, Eating, and Exercise Comparison Orientation Measure	There was a decrease in physical activity associated with an increase in sedentary behaviors, specifically spending more time watching television
Flores & Roman (2020) [30]	Peru	263	International Physical Activity Questionnaire (IPAQ)	Most of the participants did not perform physical activity as part of their routine, with the most common type of physical activity being light exercise.

**Table 3:** Body Image in university students during COVID-19.

Author (Year)	Country	Population	Instrument	Result
Márquez (2021) [25]	Colombia	16	Stunkard A and Stellar, E. 1990 caporal silhouettes	43.8% of the students perceived themselves as normal weight (Silhouette utter 6) and 25% perceived themselves as obese (Silhouette 7)
Cadena-Duarte & Cardozo (2021) [26]	Colombia	499	Physical Self Questionnaire (PSQ) adapted to the Spanish context, modified version by Moreno and Cervelló (2005)	Body image perception was better in older university students in contrast to younger ones
Baceviciene & Jankauskiene (2021) [27]	Lithuania	230	Self-reported weight (kg) and height (m); Multidimensional Body Self Relations Questionnaire (MBSRQ) appearance scales in validated version for Lithuania	BMI and satisfaction with body appearance increased significantly in women during quarantine, contrary to men who had no changes.
Bueno De Souza (2021) [28]	Brazil	294	Own elaborated questionnaire	During the period of social distancing by COVID-19, most students reported negative changes in the perception of appearance.
Keel et al., (2020) [29]	USA	90	Self-reported BMI assessment to transform values into the following categories: underweight, normal weight, overweight, and very overweight, and DSM-5 guideline for very underweight.	They found no significant changes in reported weight at Time 1 and Time 2 or BMI from Time 1 to Time 2. Despite this, there was an increase in weight description from Time 1 to Time 2.
Flores&Roman (2020) [30]	Peru	263	Body Shape Questionnaire (BSQ)	The majority of female students have no concern about their body image (47.2%).

## Data Availability

The data presented in this study are available on request from the corresponding author.
